# The draft genome of the Wisconsin Miniature Swine^TM^, a valuable biomedical research tool

**DOI:** 10.1093/g3journal/jkaf067

**Published:** 2025-04-07

**Authors:** Alex C Veith, Jennifer J Meudt, Jamie L Reichert, Jennifer M Frank, Derek M Pavelec, Bridget Ladell, James Speers, Molly Zeller, Taeyoung Shin, Joshua R Hyman, Christopher A Bradfield, Charles M Konsitzke, Dhanansayan Shanmuganayagam, C Dustin Rubinstein, Mark E Berres

**Affiliations:** University of Wisconsin Biotechnology Center, University of Wisconsin-Madison, Madison, WI 53706, USA; McArdle Laboratory for Cancer Research, University of Wisconsin School of Medicine and Public Health, Madison, WI 53706, USA; Biomedical and Genomic Research Group, Department of Animal and Dairy Sciences, University of Wisconsin-Madison, Madison, WI 53706, USA; Swine Research and Teaching Center, Department of Animal and Dairy Sciences, University of Wisconsin-Madison, Madison, WI 53706, USA; Swine Research and Teaching Center, Department of Animal and Dairy Sciences, University of Wisconsin-Madison, Madison, WI 53706, USA; University of Wisconsin Biotechnology Center, University of Wisconsin-Madison, Madison, WI 53706, USA; University of Wisconsin Biotechnology Center, University of Wisconsin-Madison, Madison, WI 53706, USA; University of Wisconsin Biotechnology Center, University of Wisconsin-Madison, Madison, WI 53706, USA; University of Wisconsin Biotechnology Center, University of Wisconsin-Madison, Madison, WI 53706, USA; Department of Surgery, University of Wisconsin School of Medicine and Public Health, Madison, WI 53706, USA; University of Wisconsin Biotechnology Center, University of Wisconsin-Madison, Madison, WI 53706, USA; University of Wisconsin Biotechnology Center, University of Wisconsin-Madison, Madison, WI 53706, USA; McArdle Laboratory for Cancer Research, University of Wisconsin School of Medicine and Public Health, Madison, WI 53706, USA; University of Wisconsin Biotechnology Center, University of Wisconsin-Madison, Madison, WI 53706, USA; Biomedical and Genomic Research Group, Department of Animal and Dairy Sciences, University of Wisconsin-Madison, Madison, WI 53706, USA; Department of Surgery, University of Wisconsin School of Medicine and Public Health, Madison, WI 53706, USA; University of Wisconsin Biotechnology Center, University of Wisconsin-Madison, Madison, WI 53706, USA; University of Wisconsin Biotechnology Center, University of Wisconsin-Madison, Madison, WI 53706, USA

**Keywords:** *Sus Scrofa;* Wisconsin Miniature Swine^TM^, WMS^TM^, genome, NF1, Genome Assembly

## Abstract

Porcine biomedical models have emerged as valuable tools in biomedical research due to their physiological, anatomical, metabolic, immunological, and genetic similarities to humans. As a result, they offer greater relevance for translational studies than rodent models. Moreover, compared to nonhuman primates, porcine models are more cost-effective, easier to manipulate genetically, and raise fewer ethical concerns. However, the conventional breeds of swine most commonly used in research have rapid growth rates, which lead to logistical challenges such as increased space requirements, making them impractical as biomedical models. The Wisconsin Miniature Swine^TM^ (WMS^TM^) was developed to address these shortcomings. The WMS^TM^ porcine model grows slower, reaching and maintaining human sizes at adulthood. The model was also specifically designed to possess more human-like physiology that allows for easy modeling of comorbidities like obesity and metabolic syndrome that affect a large portion of the human population affected by chronic diseases. Thus, WMS™ is an ideal porcine gene editing platform for modeling complex multifactorial diseases. Here, we present the first draft genome assembly representative of the WMS^TM^ line. The primary assembly was generated with ∼20× coverage of long reads from Oxford Nanopore Technologies and independently error-corrected using 23× Pacific Biosciences reads. Arima Genomics Hi-C data were used to improve contiguity. Largely congruent with the existing *Sus scrofa* genome, we also show the utility of WMS^TM^ as a model through comparisons between 2 WMS^TM^ genes and human homologs. Finally, we show the utility of genotyping by sequencing across WMS^TM^ herds. The WMS^TM^ genome generated here is highly complete and supports investigators utilizing WMS^TM^ in biomedical research.

## Introduction

Porcine models are becoming indispensable in biomedical research due to their remarkable anatomical, physiological, and genomic similarities to humans (Schomberg *et al*. 2016; Lunney *et al*. 2021; Meyerholz *et al*. 2024). These similarities are particularly pronounced in key systems such as the cardiovascular, gastrointestinal, and immune systems, making pigs highly translational models for studying human diseases and developing novel therapies (Meurens *et al*. 2012; Lelovas *et al*. 2014; Gerdts *et al*. 2015; Pabst 2020). Beyond systemic similarities, pigs also provide an optimal platform for cutting-edge imaging modalities due to their size and physiology, which closely mimic those of humans. Advanced imaging techniques, such as MRI and CT, have been effectively adapted and validated in porcine models, enabling high-resolution studies that bridge preclinical findings to clinical applications (Raval *et al*. 2022; Costine-Bartell *et al*. 2023). Moreover, swine models are increasingly being used to study aging and associated comorbidities, providing new opportunities to explore therapeutic strategies for age-related conditions (Schachtschneider *et al*. 2021; Lu *et al*. 2023). Another transformative area of research where pigs have gained prominence is xenotransplantation. The anatomical and physiological compatibility of porcine organs with human systems, combined with advances in genetic engineering to reduce immunological rejection, have positioned swine as the leading source for xenogeneic organ transplantation (Anand *et al*. 2023; Ali *et al*. 2024; Peterson *et al*. 2024). The recent successful transplantation of porcine kidneys and hearts into humans underscores the critical role of swine in addressing the global organ shortage crisis (Griffith *et al*. 2022; Porrett *et al*. 2022; Moazami *et al*. 2023).

Porcine models complement and effectively address limitations of other animal models, such as rodents and nonhuman primates (NHP), while offering unique advantages for biomedical research. Despite their high translational relevance, NHP models are constrained by limited availability, high costs, and shifting public perception regarding their use in research (Carvalho *et al*. 2018; Grimm 2023). Swine, in contrast, are more accessible, less expensive to maintain, and face fewer ethical barriers, making them a practical and scalable alternative for many research applications. The physiological and anatomical similarities between swine and humans provide a level of translational fidelity that rodent models often cannot achieve. Rodents, while valuable for genetic studies and early stage research, have shown high failure rates in translating preclinical findings to human clinical trials, particularly in areas such as cardiovascular, immune, and metabolic diseases (Gutierrez *et al*. 2015; Frangogiannis 2022).

The advent of CRISPR-Cas9 genome editing has significantly enhanced the utility of porcine models, allowing researchers to create precise genetic modifications that replicate human disease states. This capability, once a hallmark advantage of murine models, now positions swine as a powerful tool for studying genetic disorders, drug development, and regenerative medicine (Perleberg *et al*. 2018). To this end, we recently generated several porcine models of neurofibromatosis type 1 (NF1), a phenotypically complex genetic disorder in humans (Rubinstein *et al*. 2021). The *NF1*^a31/+^ swine model formed neurofibromas similar to those seen in human patients and provided novel insight into the fibroblast populations and shed light on a potential role for cancer-associated fibroblasts on the recruitment of Schwann cells to produce neurofibromas (McLean *et al*. 2023). Here, porcine models possess a clear advantage over NHP, which, despite their translational competence, are impractical for the generation of genetic models by genome editing due to their long reproductive cycles and limited number of offspring. Rhesus macaques, the most used NHP in the United States, typically reach sexual maturity at 3–5 years of age and give birth to 1 offspring per year after a gestation time of ∼165 days (Wolfensohn 2010; Campbell *et al*. 2011; Cooper *et al*. 2022; National Academies of Sciences 2023). In contrast, swine reach sexual maturity at 5–6 months of age and can give birth to 1–2 litters of 8–12+ piglets/litter per year with a gestation time of ∼114 days; many minipig breeds give birth to 4–8 piglets/litter.

Despite the translational advantages of the species, conventional breeds of swine most commonly used in research exhibit rapid growth, reaching weights of 100 kg (220 lb.) by 4 months of age and continuing to grow to 249–306 kg (550–675 lb.) at full maturity (Schomberg *et al*. 2016). This rapid growth necessitates the use of prepubertal pigs in research to maintain manageable sizes. However, using juvenile pigs does not accurately model diseases prevalent in adult human populations, as their physiological and metabolic states differ significantly from adults. Furthermore, the accelerated growth rate of conventional swine (5–6 kg increase/week) is associated with rapid tissue remodeling, which does not accurately reflect the slower tissue turnover observed in adult humans. This discrepancy can complicate the translatability of research findings, particularly in studies involving tissue regeneration, wound healing, and chronic disease progression. Thus, miniature (“minipig”) breeds have received greater attention recently (Gutierrez *et al*. 2015). These breeds better grow slower and match human size at maturity while also reducing required husbandry resources. They also exhibit a greater physiological similarity to humans than conventional breeds. For example, higher similarity between minipig liver enzymes and those of humans, indicating a greater translational value in pharmacokinetic studies, has been noted (Suenderhauf and Parrott 2013).

**Fig. 1.**
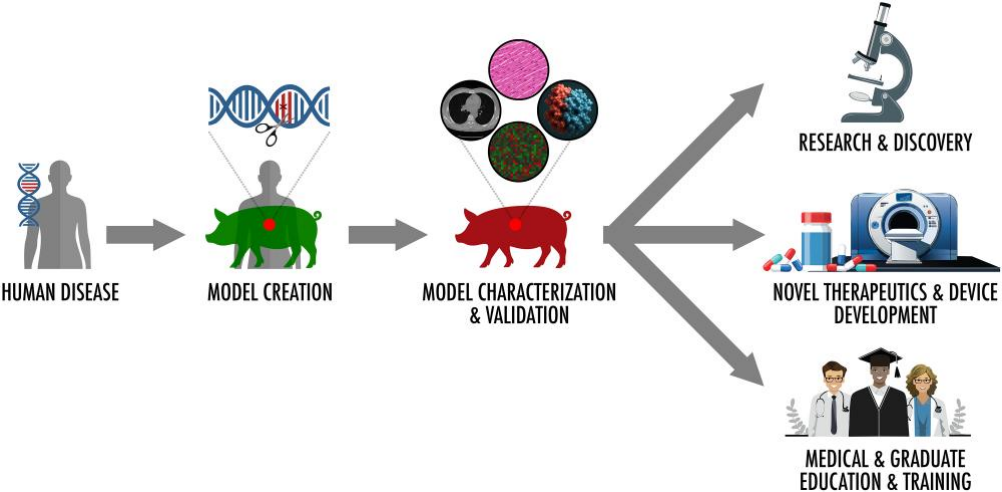
The WMS^TM^, developed at UW-Madison, is an important translational platform for creation of genetic models. It has unique value in preclinical and translational research and as an education and training tool.

The Wisconsin Miniature Swine^TM^ (WMS^TM^) was developed at the University of Wisconsin-Madison to provide the many advantages of minipigs, including age-related changes in genes associated with obesity and lipid metabolism comparable to humans (Fig. 1). The WMS^TM^ breed reaches puberty at about 5–6 months of age with a body weight of ∼27–32 kg (59–70 lb.) and can be maintained at human sizes [68–91 kg (150–200 lb.)] during adulthood (Schomberg *et al*. 2016). In contrast, while conventional breeds reach puberty at the same age, they typically weigh 110–135 kg (242–297 lb.). The WMS^TM^ is derived from the once widely used Rapacz-Familial Hypercholesteremia (FH) mini swine, also developed at the university, which carried a clinically relevant mutation in the low-density lipoprotein receptor (LDL-R) associated with high circulating blood cholesterol (Rapacz *et al*. 1994; Hasler-Rapacz *et al*. 1998). The Rapacz-FH line, a hybrid of 37 pig breeds throughout Europe and North America, was bred to Guinea Hogs for 1 generation and interbred twice to produce unique WMS^TM^-WT (wildtype) and WMS^TM^-Familial Hypercholesteremia (WMS^TM^-FH) lines. WMS^TM^-WT was also previously published as WMS^TM^-N (normal) (Namous, Krueger, *et al*. 2023; Namous, Strillacci, *et al*. 2023). The WMS^TM^-FH line harbors the LDR-R mutation, while the WMS^TM^-WT does not (Schomberg *et al*. 2016). Thus, WMS^TM^-FH develops human-like complex atherosclerotic lesions in coronary arteries and other arterial beds that display necrotic core, calcification, and neovascularization (Namous, Strillacci, *et al*. 2023). Moreover, we have demonstrated that, like humans, the model develops neointimal restenosis in coronary arteries after stent implantation, unlike conventional swine breeds (Schomberg *et al*. 2016). Both lines have been invaluable in biomedical research (Lin *et al*. 2018; Miranpuri *et al*. 2018; Horton *et al*. 2020; Johnson *et al*. 2021; Schachtschneider *et al*. 2021; Wallat *et al*. 2021; Wuschner *et al*. 2021, 2022a, 2022b; Eix *et al*. 2022; Hanna *et al*. 2022; Johnson *et al*. 2022; Pirasteh *et al*. 2022; Lu *et al*. 2023; Flakus *et al*. 2023a, 2023b, 2023c; Namous, Krueger, *et al*. 2023; Namous, Strillacci, *et al*. 2023; De La Cruz *et al*. 2024). Here, we present the draft genome of the WMS^TM^ to establish the genomic toolkit needed to broaden the utility of these lines and accelerate biomedical advancements using porcine models.

## Materials and methods

### Herd maintenance

The animal protocols related to this work were approved by the Institutional Animal Care and Use Committee at the University of Wisconsin-Madison in accordance with the *Guide for the Care and Use of Laboratory Animals* and the Animal Welfare Act. The WMS^TM^-WT and WMS^TM^-FH lines are housed at the University of Wisconsin Swine Research and Teaching Center (Arlington, WI), a specific pathogen-free facility. Both lines receive the same diet, formulated with essential macronutrients and micronutrients to meet their requirements for normal growth and development.

### Sample preparation

A whole blood sample was collected from a representative adult male WMS^TM^-WT pig (“Benny;” Pig ID: 18-4274-3; Age: 1 yr, 6 months, 25 d) for the purpose of genome assembly. The boar used in this study was frequently used for breeding in the WMS^TM^-WT herd. Additional blood samples were obtained from both the WMS^TM^-WT and WMS^TM^-FH lines to conduct genotyping by sequencing (GBS) analysis. These collected samples were submitted to the University of Wisconsin-Madison Biotechnology Center DNA Sequencing Facility for processing. High molecular weight (HMW) DNA (25 µg) was extracted from leukocytes using the Circulomics Nanobind CBB Big DNA kit (Pacific Biosciences, Menlo Park, CA, USA). Nucleic acid concentrations were quantified with the Quant-iT^TM^ PicoGreenTM dsDNA and Qubit^TM^ dsDNA High Sensitivity kits (ThermoFisher Scientific, Waltham, MA, USA) used for Oxford Nanopore and Pacific biosciences (PacBio) runs, respectively. The concentrations (A260/A280) and purity (A260/A230) of DNA were determined using a NanoDrop^TM^ One spectrophotometer (ThermoFisher Scientific, Waltham, MA, USA). Finally, the sizing and quality of diluted samples were assessed using the FemtoPulse System (Agilent Technologies, Madison, WI, USA). The average read size, between 40,000 and 508,400 bp, was 244,172 (bp).

### Whole genome sequencing

Whole genome sequencing was performed using 2 third-generation sequencing platforms and a genome-wide proximity ligation (Hi-C) method, resulting in an error-corrected, chromosome-scale assembly of the WMS™ genome.

#### Oxford nanopore technologies sequencing

Oxford nanopore technologies (ONT) sequencing was utilized to generate the initial reads used for assembling the WMS^TM^ draft genome. Sequencing libraries were prepared from 3 µg of input DNA using the SQK-LSK109 ligation sequencing kit (Oxford Nanopore Technologies, Oxford, UK) and quantified with the Qubit^TM^ dsDNA High Sensitivity Kit (ThermoFisher Scientific). The sequencing was performed on 5 MinION flow cells processed on a GridION instrument (Oxford Nanopore Technologies).

#### PacBio sequencing

PacBio sequencing was utilized to obtain highly accurate reads to error correct (“polish”) the assembly produced by the ONT reads. A PacBio HiFi SMRTBell library was prepared from 5 µg of input DNA according to PN 101-693-800 Version 01 (Pacific Biosciences, Menlo Park, CA, USA), with the following modifications: (1) shearing with gTUBES (Covaris, LLC, Woburn, MA, USA) and (2) size selection with BluePippin (Sage Sciences, Beverly, MA, USA). The quality of the library was determined by the FemtoPulse System (Agilent Technologies, Madison, WI, USA), and the Qubit^TM^ dsDNA High Sensitivity Kit (ThermoFisher Scientific, Waltham, MA, USA) was used to quantify the library preparation. PacBio sequencing was performed on a Sequel I instrument (Pacific Biosciences, Menlo Park, CA, USA) with 4 PacBio 4 M SMRT cells.

#### Arima Hi-C sequencing

Hi-C sequencing was utilized to scaffold the error-corrected ONT draft genome assembly. Sample processing, crosslinking, and Hi-C library preparation were performed according to the manufacturer's instructions for the Arima HiC v1 kit for Mammalian Blood (Material Part Number: A410030, Document Part Number: A160104 v01; Arima Genomics, Carlsbad, CA, USA) and the KAPA Hyper Prep Kit (Roche, Basel, CH) with the following modifications: during PBMC isolation, phosphate-buffered saline (PBS) was used for the first step as directed but subsequent steps used fetal bovine serum (FBS) and adapter ligation was increased from 15 min to 1 h. Library quality was assessed with an Agilent TapeStation (Agilent Technologies) and quantified with a Qubit^TM^ dsDNA High Sensitivity (ThermoFisher Scientific) kit. The resulting Illumina TruSeq library was sequenced as paired-end (2 × 150 bp) reads on a NovaSeq6000 (Illumina, Inc., San Diego, CA, USA).

### Genome assembly

ONT reads meeting a minimum Q-value of 10 (phred scale) were size- and chimera-filtered (minimum length = 10,000 bp) to approximately 23× coverage with Filtlong v0.2.0 (https://github.com/rrwick/Filtlong) and Porechop v0.2.4 (https://github.com/rrwick/Porechop). Filtered reads were assembled with Flye v2.5, enforcing a 10,000 bp overlap minimum (Kolmogorov *et al*. 2019). Assembled sequences <10,000 bp were filtered from the draft assembly. Approximately 20× PacBio subread coverage was used with GCpp v1.0.0-1807624 (https://github.com/PacificBiosciences/gcpp) to independently error-correct the ONT-based Flye assembly. The resulting error-corrected haploid (pseudohaploid) assembly was deduplicated (*i.e.* identification of syntenic elements, keeping only 1 element) with purge_haplotigs commit be7e44b (https://bitbucket.org/mroachawri/purge_haplotigs/src/master/) (Roach *et al*. 2018). Juicer v1.6.2 (https://github.com/aidenlab/juicer) and 3D-DNA v180114 (https://github.com/aidenlab/3d-dna) were used to scaffold the deduplicated, error-corrected assembly with the Hi-C data (Durand *et al*. 2016; Dudchenko *et al*. 2017). We assessed the quality of our genome assembly using BUSCO v5.7.0 with mammalia_odb10 (https://busco.ezlab.org/) and QUASTv5.0.2 (https://quast.sourceforge.net/) (Gurevich *et al*. 2013; Manni *et al*. 2021).

### Gene identification

Putative genes in the WMS^TM^ draft genome were identified using Liftoff v1.6.3 (https://github.com/agshumate/Liftoff) with default parameters against the reference *S. scrofa* Ensembl *a*nnotation for Sscrofa11.1 (The Swine Genome Sequencing Consortium, 2017, Genome assembly Sscrofa11.1, NCBI, GCF_000003025.6), genebuild 2022-02 (Shumate and Salzberg 2021).

### Gene diversity analysis

The draft WMS^TM^ genome and annotation files produced here were interrogated for the *NF1* (neurofibromin) and *AHR* (aryl hydrocarbon receptor) genes. For each putative WMS^TM^ gene, we extracted the entire annotated gene sequence and aligned it to the *S. scrofa* reference coding sequence (CDS) using the align sequences feature in SnapGene^TM^ Version 7.2.1 (GSL Biotech, Boston, MA, USA). The reference sequence was selected based on Ensembl's canonical distinction. The aligned CDS from the WMS^TM^ gene were translated and aligned to the respective translated CDS in *S. scrofa* as well as the canonical human CDS using SnapGene^TM^ software. The BLOSUM62 similarity matrix was used to delineate similar amino acids. The WMS^TM^ and human amino acid sequences were then input into AlphaFold 3 for structural predictions using the same seed sequence (Abramson *et al*. 2024). The structural predictions from WMS^TM^ and human proteins were aligned using PyMOL (The PyMOL Molecular Graphics System, Version 3.0.5 Schrödinger, LLC.)

### Genotype by sequencing

Genetic variation across the WMS^TM^-WT and WMS^TM^-FH herds was assessed using genotype-by-sequencing (GBS), a method licensed to the UW-Madison Biotechnology Center by KeyGene N.V. (Wageningen, NL). GBS libraries were prepared as previously described with minimal modification (Elshire *et al*. 2011). Whole blood was collected from individuals across both herds, and DNA was isolated with a QIAamp 96 DNA QIAcube HT Kit. One hundred-fifty nanograms of purified DNA was then digested by the restriction enzymes NsiI and MspI. Barcoded adaptors compatible with Illumina sequencers were added to restricted-DNA by ligation using T4 ligase (New England Biolabs, Ipswich, Mass.). Three plates, each containing up to 96 adapter-ligated samples, were each pooled and amplified to generate a sufficient sequencing template. Adapter dimers were removed by SPRI bead purification. The quality and quantity of the finished libraries were assessed using the Agilent TapeStation (Agilent Technologies, Inc., Santa Clara, Calif.) and Qubit^TM^ dsDNA HS Assay Kit (Life Technologies, Grand Island, N.Y.), respectively. Libraries were sequenced on a NovaSeq6000 S2 platform (Illumina, Inc., San Diego).

The University of Wisconsin Bioinformatics Resource Center (UWBRC) completed quality control, sequence alignment, and SNP calling on GBS data. Adapters, low-quality bases, and primers were trimmed from reads to obtain a Phred score of 20 via the trimming software Skewer (v0.2.2.b). Reads that were too short to be used were also discarded. (Jiang *et al*. 2014). The Tassel pipeline (version 2) was used to process sequence reads to single nucleotide polymorphism (SNP) genotypes (Glaubitz *et al*. 2014). Briefly, demultiplexed reads were aligned to the WMS^TM^ draft genome using Bowtie2 (Langmead and Salzberg 2012). The Tassel pipeline was then used to identify potential tags in the aligned SAM files compared to the WMS^TM^ reference genome, outputting a VCF file. Filtering was performed on VCF files to include only biallelic sites and a minor allele frequency (MAF) ≥ 0.05. SNP coverage and coverage per sample were then calculated. The population-wide inbreeding coefficient F was calculated with VCFtools (v.0.1.16) (Danecek *et al*. 2011).

Sample relationships were assessed using principal component analysis (PCA), and a bifurcating topology was generated using maximum likelihood. Briefly, for PCA analysis, SNPs were filtered by those with information at ≥60% of samples, and samples with ≤80% of pre-filtered single nucleotide polymorphisms (SNPs) were excluded. Beagle v4.1 was used to perform whole genome phasing and to impute missing data (Browning and Browning 2007, 2016). A mid-point rooted topology was created from the VCF data using RAxML and visualized with itol v6.9.1 (https://itol.embl.de/) (Stamatakis 2014; Huerta-Cepas *et al*. 2016; Ortiz 2019; Letunic and Bork 2024).

## Results and discussion

### Genome assembly

We anticipated generating a WMS^TM^ genome assembly of approximately 2.5 Gb based on its expected similarity to *S. scrofa* (Sscrofa11.1). The reference genome assembly of Sscrofa11.1 was completed by adding Y chromosome sequences from other sources (GCA_900119615.2) because TJ Tabasco (Duroc 2–14) was female. The resulting reference genome sequence was termed Sscrofa11.1 and deposited in the public sequence databases (GCA_000003025.6), which is the reference used in this work (Warr *et al*. 2020). Oxford Nanopore (ONT) sequencing was chosen for the initial pass of the genome assembly due to its ability to generate extremely long reads and produce highly contiguous data (Lu *et al*. 2016). Sequencing data generated from 5 ONT MinION flow cells produced an input read length of approximately 48 Gb, achieving an expected coverage of about 20X. Using the Flye assembler, we generated a draft WMS^TM^ assembly consisting of 1,215 contigs exhibiting a mean coverage of 17X.

One characteristic of the ONT sequencing platform is a relatively high error rate, which necessitates additional error correction (Delahaye and Nicolas 2021; Wang *et al*. 2021). While overlap layout consensus (OLC) tools are available to error-correct assemblies using the same reads input into the assembly, we opted to perform error correction independently with PacBio reads (Chen *et al*. 2021). We obtained a total read length input of 58 Gb from 4 SMRT cells (approximately 23× coverage). Following error correction, the number of contigs was reduced by 16.6% from 1,215 to 1,013. The L50 of the error-corrected assembly was 22 contigs exhibiting an N50 of 36.751 MB (max contig length = 125.705 MB).

Regions of high heterozygosity can lead Flye, as well as other genome assemblers, to produce assemblies that exceed the expected haploid genome size. When allelic sequences reach a threshold level of nucleotide divergence, standard assembly algorithms may treat these regions as distinct sequences, generating separate contigs instead of a single, merged haplotype contig. This phenomenon reflects a limitation in the assembler's ability to reconcile high-divergence alleles within a unified assembly, which may lead to an over-representation of allelic regions in the final assembly output. We used Purge Haplotigs to mitigate this over-representation by identifying and removing redundant contigs derived from allelic variation, thereby refining the assembly to better reflect the true haploid genome structure better. Purge Haplotigs typically generates 2 distinct peaks in the k-mer distribution plot, corresponding to homozygous and heterozygous regions in assemblies containing syntenic haplotigs. However, when applied to the WMS^TM^ draft genome, we observed only a single peak with no discernible shoulder peaks. This outcome may reflect biologically low heterozygosity (*i.e.* identical alleles at most loci) or insufficient and/or uneven sequencing coverage, which could obscure the k-mer differences between homozygous and heterozygous regions. The software aggressively removed even large contigs when we applied Purge Haplotigs using permissive cutoffs. Given the existing low contig count, the N50 metric capturing the 20 expected chromosomes (MUMmer4 analysis), and the risk of false positives, we opted not to apply Purge Haplotigs to the draft assembly.

Hi-C data was incorporated to enhance the contiguity of the genome assembly, following the approach described elsewhere (Burton *et al*. 2013). We generated approximately 400 M 2 × 150 bp reads using Illumina sequencing. Systematic integration of the Hi-C data yielded a marked improvement in contiguity. However, comparison with the Sscrofa11.1 reference genome using MUMmer4 often revealed structural inconsistencies, including large- and small-scale mis-joins, sub-chromosomal inversions, and inter-chromosomal translocations (Marcais *et al*. 2018). These issues were likely exacerbated by regions of lower sequence coverage, particularly evident on the X and Y chromosomes, which had an average coverage of approximately 10x. While we expect some inherent structural variation in the WMS^TM^ genome, the overall synteny is consistent with Sscrofa11.1, comprising 18 autosomes and the X and Y sex chromosomes. To address potential misassembles, larger contigs generated with Hi-C data were manually reviewed. This involved aligning the original Oxford Nanopore reads to each contig for verification. Subsequently, CRAQ v1.0.9 (https://github.com/JiaoLaboratory/CRAQ) was used to detect large-scale structural errors, termed clip-based structural errors (CSEs) (Li *et al*. 2023).

**Fig. 2.**
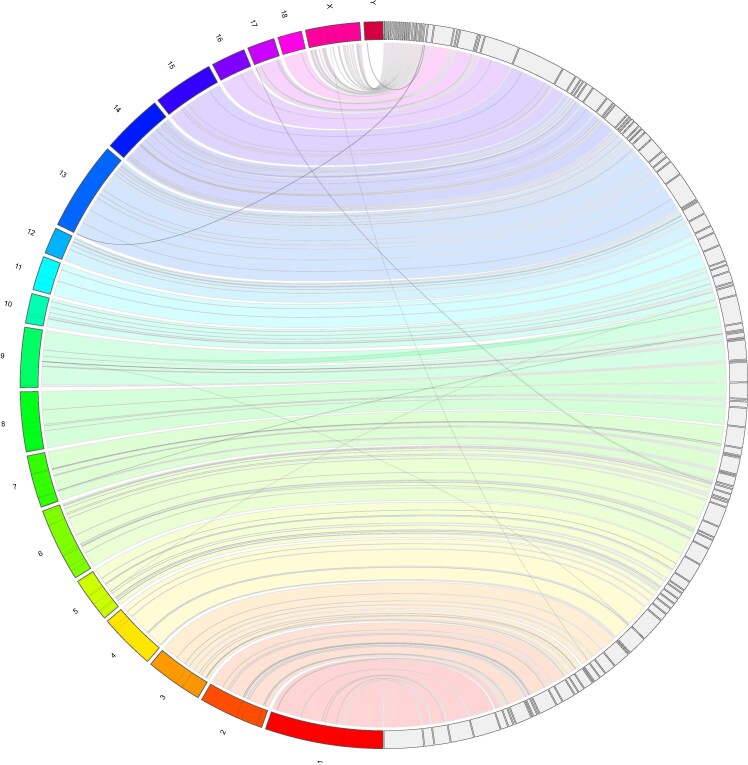
Visualization of WMS^TM^ and *S. scrofa* syntenic identity. The *S. scrofa* scaffold is represented on the left hemisphere compared to the WMS^TM^ scaffold, which is depicted on the right hemisphere. The Circos-based plot links the reference chromosomes to the scaffolds generated in the WMS^TM^ assembly, with each chromosome uniquely shaded. We see strong similarity between the 2 genomes but do note structural differences.

Contigs were split at locations where significant clipping in the Oxford Nanopore reads indicated the presence of CSEs. In addition to errors introduced by the assembler, these structural inaccuracies may also result from incomplete DNA digestion during Hi-C preparation, erroneous ligation of DNA fragments, or misassignment of long-range contacts during scaffolding, which can misrepresent true genomic architecture. After manual inspection, JupiterPlot v1.1 (https://github.com/JustinChu/JupiterPlot) was used to create a syntenic comparison of WMS^TM^ to the Sscrofa11.1 reference genome (Fig. 2). The final draft assembly was considered complete following manual curation, with the assembly statistics summarized in Table 1.

**Table 1.** Final assembly information.

Total length (bp)	2,517,610,705
Total length > 50,000 bp	2,511,032,328
# Contigs	1,022
# Contigs > 50,000 bp	729
Largest contig (bp)	108,923,112
N50 (bp)/L50	35,546,377/25
N90 (bp)/L90	2,592,705/102
auN (bp)	36,668,315.6
GC	42.15%
# N's per 100 kbp	0.19

To evaluate the quality of our assembly, we performed a BUSCO assessment of conserved single-copy orthologous sequences. The WMS^TM^ draft assembly achieved a BUSCO score of 98.2% ([S:97.9%, D:0.3%], F:1.0%, M:0.8%), indicating that we have a highly complete assembly. Notably, the detection of only 27 duplicated BUSCOs out of 9,226 indicates a low prevalence of haplotigs, supporting the decision that haplotig purging was not strictly necessary. It remains possible that false collapses affected the number of identified duplications. The BUSCO assessment of the assembly generated here surpasses the BUSCO scores for existing *S. scrofa* reference genomes (Sscrofa11.1 = 93.8% and USMARCv1.0 = 93.1%) (Warr *et al*. 2020). Overall, our data indicates that the draft WMS^TM^ genome is a highly complete genome assembly.

At the time of our analysis, Porechop was the only tool available that provided comprehensive functionality for both demultiplexing and adapter trimming. While newer tools such as Guppy (Guppy_barcoder) have since improved and now supersede Porechop, they were still under development during the initial stages of our study. Specifically, Porechop outperformed early versions of Guppy in terms of accuracy and reliability for demultiplexing and trimming, as evidenced by our own internal benchmarks at the time. The same is true for sequencing error rates, which have been reduced by refinements in chemistry and algorithms. Our analysis focused on ensuring consistency and reliability with the tools available at the time the study was conducted. While it is true that reanalyzing data with updated tools can provide refinements, our approach ensures the reproducibility of our methods as initially performed. Moreover, the tools used were state-of-the-art for their respective timeframes, reflecting the quality of the data generated and the robustness of our comparisons.

### Annotation

The WMS^TM^ line was created from a hybrid of approximately 37 *S. scrofa* breeds, predominately Guinea Hog (*Sus scrofa domesticus*), and therefore, we expected high identity with the reference *S. scrofa* genome. To analyze this, we employed Liftoff, an annotation lift-over tool that maps gene features from closely related species onto a target genome (Shumate and Salzberg 2021). When applied to WMS^TM^, the lift-over annotation resulted in a 97.76% match with Sscrofa11.1 annotations with an alignment coverage and sequence identity ≥50%. The gene features in the *S. scrofa* genome used in our analysis included 20,306 annotations, of which we identified 19,853 matches (96.4%). This number reflects similar gene counts identified in other swine models and indicates high concordance between the gene features of WMS^TM^ and *S. scrofa*. Notably, 93% of the 453 genes that were not mapped by the Liftoff analysis have no assigned function. Our study has demonstrated that Liftoff provides robust and accurate annotations, meeting the objectives of this research by providing a high-quality functional annotation.

### Comparison to *S. scrofa*

The WMS^TM^ assembly generated here exhibited a strong identity with the Sscrofa11.1 genome build (Fig. 2). The cross-genome analysis identified several small structural variations between the WMS^TM^ and *S. scrofa* genomes. There was also a prominent inversion on chromosome 9 that persisted throughout our analysis. This inversion was present with and without Hi-C assembly information, and our data strongly suggests that this is a *bona fide* difference from Sscrofa11.1. It is likely that structural variations exist due to both assembly errors and true genetic variation. Additional work will be needed to validate the WMS^TM^ genome, including potential structural variations (Yang *et al*. 2024).

### Gene-level comparison

To illustrate the utility of WMS^TM^ as a model for human disease or discovery, we compared sequence variation between WMS^TM^, *S. scrofa,* and human *NF1* and *AHR* genes. One active area of interest to our group is the use of swine models in NF1 (Rubinstein *et al*. 2021; McLean *et al*. 2023). In our previous research, we utilized larger swine breeds, which limited the number of animals we could maintain. To demonstrate the utility of the WMS^TM^ as a biomedical research platform, we compared the gene annotations of the *NF1* and *Ahr* genes WMS^TM^ to the *S. scrofa* and human reference transcripts to determine their similarity.

**Fig. 3.**
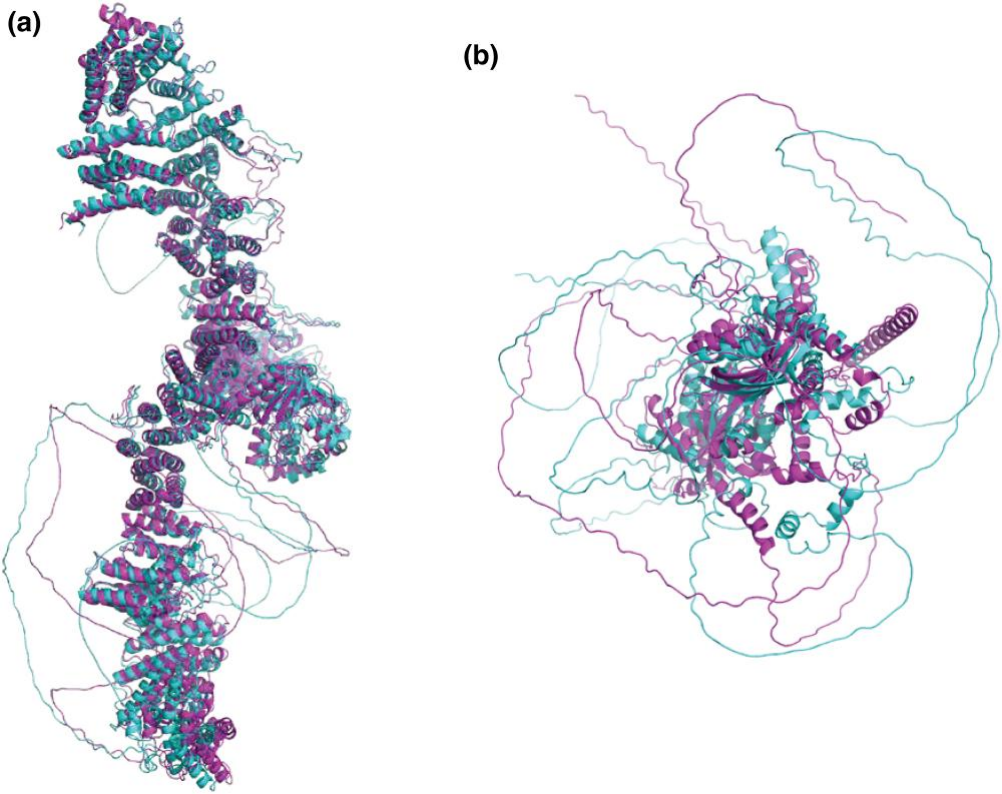
Structural comparison of WMS^TM^ and human NF1 and AHR proteins. a) Alignment of the predicted structures of the WMS^TM^ (magenta) and human (cyan) NF1 proteins. We see strong structural similarity between both proteins, strongly supporting porcine models, and particularly WMS^TM^, as a platform for NF1 research. b) Alignment of the predicted AHR structures of WMS^TM^ (magenta) and human (cyan). 360-degree movie clips are included in the supplemental files. Relative sizes of NF1 and AHR proteins are not depicted to scale.

The annotation lift-over identified a ∼265kb-long putative *NF1* gene in the WMS^TM^ genome that matched the *S. scrofa NF1* gene (ENSSSCG00000017748). The Ensembl canonical CDS for the *NF1* gene CDS (ENSSSCT00000093848.2) produces a 2,839-long amino acid sequence when translated. The ENSSSCT00000093848.2 was successfully aligned to the putative WMS^TM^  *NF1* gene to produce the WMS^TM^  *NF1* CDS, encoding a 2,840-long amino acid. Local alignment of the amino acid sequences showed a sequence identity match of 99.96%, differing by a single amino acid insertion (Pro771_Ala772insThr) in the WMS^TM^ NF1 sequence. Comparison of the WMS^TM^ NF1 amino acid sequence to the human homolog (ENST00000358273.9) revealed a 99.12% (2817/2842) identity match and an additional 10 positions in which they shared similar amino acids (99.47% similarity match). Structural prediction and alignments show a strong similarity, reflected by a root mean square deviation of 2.114 Å (Fig. 3a). The potential structural differences tended to be localized to areas that are predicted with low confidence. This was expected, as *NF1* has been implicated in regulating tumorigenesis, and others have demonstrated strong sequence similarities between human and murine homologs (Bernards *et al*. 1993; Jouhilahti *et al*. 2011).

In contrast, the AHR is a ligand-dependent transcription factor that is highly conserved yet exhibits significant sequence variation between species (Hahn 2002; Hahn *et al*. 2017). We have recently described a “xenokine” model in which AHR ligands regulate physiology (Avilla *et al*. 2020). Swine have been used to isolate endogenous AHR ligands, and we hypothesize that WMS^TM^ may be a useful model to study the role of AHR in physiology (Song *et al*. 2002). The annotation lift-over identified a ∼45 kb-long putative *AHR* gene in the WMS^TM^ genome that corresponds with the *S. scrofa AHR* gene (ENSSSCG00000030484). Alignment of the *S. scrofa* canonical *AHR* CDS (ENSSSCT00000031031.4) to the WMS^TM^ genome produced a CDS encoding an 844-long AHR amino acid that shared a 98.35% identity match to the *S. scrofa* AHR amino acid sequence. This level of variability is consistent with previous findings between *Ahr* alleles between different lines of mice (Poland *et al*. 1994). To this end, the WMS^TM^ AHR amino acid sequence shared an 81.66% identity match to the human AHR (699/856 positions), with an additional 61 positions sharing similar amino acids (88.79%). The predicted structures of both the WMS^TM^ and human AHR proteins were aligned, as shown in Fig. 3b.

Porcine models hold substantial translational potential due to their remarkable physiological and anatomical similarities to humans, coupled with their susceptibility to precise genetic modifications (Lunney *et al*. 2021; Rubinstein *et al*. 2021). The high-quality draft WMS^TM^ genome presented here is a valuable resource for comparative genomic analyses and the informed design of targeted mutations for modeling human diseases. Our analysis demonstrates that the WMS^TM^  *NF1* allele exhibits significant sequence and likely functional homology to the human ortholog. This finding emphasizes the utility of the WMS^TM^ genome as a robust platform for studying NF1-related pathologies, allowing greater access to the molecular and phenotypic mechanisms underlying NF1 disorders in a physiologically relevant model system. Moreover, the availability of this genomic resource is anticipated to facilitate broader comparative genomic studies across species and accelerate the development of innovative therapeutic strategies.

### Genotyping by sequencing

GBS is a cost-effective and high-quality approach commonly used in agricultural settings to assess genetic variation at the population level (Wang *et al*. 2020). GBS is a reduced-representation sequencing approach, leveraging restriction-enzyme accessible, variant-harboring targets to define population structure and conduct genome-wide association analyses (Elshire *et al*. 2011; Scheben *et al*. 2017; Hess *et al*. 2020).

**Fig. 4.**
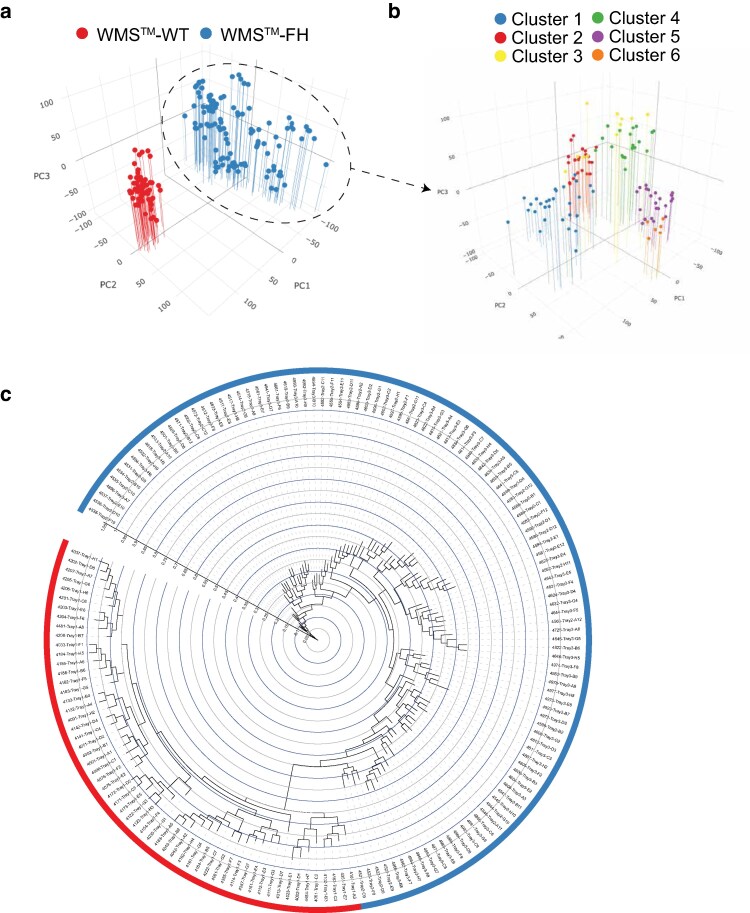
Population dynamics of WMS^TM^. a) Principal component analysis of the WMS^TM^-WT (red) and WMS^TM^-FH (blue) herds. There is a clear separation between the WMS^TM^-WT and WMS^TM^-FH herds, likely due to the association with the FH allele. b) To provide improved resolution of a single herd, the WMS^TM^-FH herd was reanalyzed separately. Removing the effect caused by the FH allele allows for finer segregation of subpopulations, which are colored independently. c) GBS phylogenetic tree indicating the relationship between all individuals across the WMS^TM^-WT and WMS^TM^-FH herds. Samples from each herd were identified by the outer color band (color from panel a).

For our GBS analysis, we collected DNA from 57 individual animals in the WMS^TM^ herd and 113 individual animals in the WMS^TM^-FH herds, representing the entire herd population at that time (170 total). We generated an average of 2.5 M demultiplexed reads per sample, which provided 186,903 total SNPs, with 92,510 meeting our filtering criteria. Ordination analysis of biallelic SNPs revealed robust segregation of unique subpopulations, with the first 3 principal components explaining 65% of the variance in the dataset. Notably, a single principal component clearly distinguished between WMS^TM^-WT and WMS^TM^-FH samples (Fig. 4a). This result was expected, as these herds were selectively bred to retain the FH allele and were maintained separately, reflecting their genetic divergence. A PCA biplot of all samples further highlighted the primary differences between the WMS^TM^-WT and WMS^TM^-FH herds. To explore potential subpopulations within the WMS^TM^-FH herd, we reanalyzed this group independently using PCA in conjunction with a k-means silhouette procedure to define groups (Fig. 4b). Without the confounding effect of FH allelic discrimination, this analysis provided finer resolution and revealed distinct subpopulations within the WMS^TM^-FH herd. Lastly, we constructed a bifurcating topology with a maximum likelihood approach to detail the relationships among all samples (Fig. 4c).

The FIS (F) of 170 individuals based on filtered GBS data was 0.38. At this level, it highlights a substantial deficit in heterozygosity, indicating moderate to high inbreeding within the population. In other words, there is a significant deficit of heterozygous individuals in the WMS^TM^ herd compared to what is expected under Hardy-Weinberg equilibrium. This suggests that individuals are more genetically similar than expected, which could be due to (1) Inbreeding and/or (2) population substructure (Wahlund effect; a mixed population made up of subpopulations can show reduced heterozygosity when treated as one). Our data indicate that both issues are likely to be present in the WMS^TM^ herd. FIS in domestic swine, especially commercial lines, is likely to be much lower, in the range of 0.1 to 0.3 (Tang *et al*. 2013). We anticipate that inbred populations used in research or specialized breeding programs exhibit higher F values (>0.30) (Gomez-Raya *et al*. 2015).

The application of GBS in managing the WMS^TM^ herd offers 2 significant advantages. First, as a newly established line, the WMS^TM^ herds (WMS^TM^-WT and WMS^TM^-FH) likely exhibit some magnitude of founder effect. GBS allows for a more comprehensive assessment of genetic diversity, which will enhance our understanding of population-level dynamics. Our findings indicate that, although the WMS^TM^ animals display overall genetic similarity, there is evidence of structured genetic diversity within both the WMS^TM^-WT and WMS^TM^-FH herds as well as within the WMS^TM^-FH herd. Second, GBS helps associate specific traits with genetic loci. While inbred animal models have been a staple in biomedical research for decades, growing evidence suggests that outbred models may offer superior translational relevance (French *et al*. 2015; Tuttle *et al*. 2018). The observed genetic segregation between the WMS^TM^-FH and WMS^TM^-FH samples underscores the feasibility of using GBS to detect and track genetic variation related to phenotypes across a population, advancing the utility of outbred models in research.

## Conclusions

In this study, we present the draft genome of the WMS^TM^, a minipig line developed as a novel tool for biomedical training and translational research. Our assembly achieved high completeness, with a BUSCO score exceeding that of the current *S. scrofa* reference. We also successfully annotated the genome. As expected, the WMS^TM^ genome exhibits a strong syntenic identity with the *S. scrofa* genome. To illustrate its research utility, we compared the WMS^TM^ and human *NF1* and *AHR* genes, underscoring the potential of WMS^TM^ as a model for studying NF1 disease mechanisms and advancing precision therapeutics. Additionally, our population-level analysis of the WMS^TM^ herd demonstrates clear genetic structuring between WMS^TM^-WT and WMS^TM-^FH individuals, establishing a foundation for genetic monitoring and trait association within this unique animal model.

While this study focuses on the genome of WMS^TM^, we recognize that additional analysis will be needed to assess genetic and epigenetic drivers of disease. For example, WMS^TM^ inherently possess DNA methylation patterns of accelerated biological aging, including increased susceptibility to cardiovascular disease, that parallels epigenetic signatures in humans associated with body mass index (Schachtschneider *et al*. 2021). These findings suggest that the WMS^TM^ model may serve as a translational bridge for studying disease mechanisms relevant to human health. Future studies will extend this work by incorporating comparative analysis of immune-related gene variants and cancer-associated mutations. Such investigations could enhance the utility of this genome assembly for the broader biomedical community, particularly in the context of genome editing applications and disease modeling. By exploring these avenues, we hope to provide further insights into the genetic underpinnings of chronic diseases and contribute to the development of improved translational models for aging and disease research.

## Data Availability

The WGS has been deposited at GenBank under the accession JBJJOT000000000.1. The version described in this paper is JBJJOT010000000 under NCBI BioProject PRJNA1190099. The sequencing data used to assemble the genome as well as the GBS data are also associated with NCBI BioProject PRJNA1190099. The unannotated genome, GFF3 annotation, and supplemental data files are available at figshare at https://doi.org/10.6084/m9.figshare.27904893.v1.
