# Correction to: The draft genome of the Wisconsin Miniature Swine^TM^, a valuable biomedical research tool

**DOI:** 10.1093/g3journal/jkaf211

**Published:** 2025-09-30

This is a correction to: Alex C Veith, Jennifer J Meudt, Jamie L Reichert, Jennifer M Frank, Derek M Pavelec, Bridget Ladell, James Speers, Molly Zeller, Taeyoung Shin, Joshua R Hyman, Christopher A Bradfield, Charles M Konsitzke, Dhanansayan Shanmuganayagam, C Dustin Rubinstein, Mark E Berres, The draft genome of the Wisconsin Miniature Swine^TM^, a valuable biomedical research tool, *G3 Genes|Genomes|Genetics*, Volume 15, Issue 6, June 2025, jkaf067, https://doi.org/10.1093/g3journal/jkaf067

In the originally published version of this manuscript, Jennifer M Frank’s present affiliation was omitted. This error has been corrected.

